# Cervical intraepithelial neoplasia grade 1 and long-term risk of progression and treatment

**DOI:** 10.1371/journal.pone.0320739

**Published:** 2025-04-23

**Authors:** Ingrid Baasland, Tone Bjørge, Birgit Engesæter, Ameli Tropé, Signe Opdahl

**Affiliations:** 1 Section for Cervical Cancer Screening, Cancer Registry of Norway, Norwegian Institute of Public Health, Oslo, Norway; 2 Department of Public Health and Nursing, Norwegian University of Science and Technology, Trondheim, Norway; 3 Department of Global Public Health and Primary Care, University of Bergen, Bergen, Norway; University of Arkansas for Medical Sciences, UNITED STATES OF AMERICA

## Abstract

**Background:**

Cervical intraepithelial neoplasia grade 1 (CIN1) is often managed by active surveillance, as the risk of progression to high grade lesions is reported to be low, but long-term data is sparse. To inform management of CIN1, we estimated risk of progression and occurrence of treatment in women attending a cervical cancer screening program.

**Methods:**

We used nationwide, registry data on all women aged 25–69 years attending the Norwegian Cervical Cancer Screening Program in 2002–2019. The eligible source population was women with at least one cytology registration (n = 1,771,876). Women with a histologically confirmed, first CIN1 diagnosis were included (n = 26,130) and followed for detection of CIN2+, defined as histologically confirmed CIN2, CIN3, adenocarcinoma *in situ* (AIS), or cervical cancer. CIN3+ was defined as CIN3, AIS, or cervical cancer. Treatment included both excision and ablation.

**Results:**

Overall, the cumulative incidence of CIN2+ increased to 9.5% (95% confidence interval (CI), 8.8 to 9.5) during the first year and to 19.0% (95% CI, 18.4–19.5) during the first five years. For women with high-grade cytology, 5-year cumulative incidence reached 26.0% (95% CI, 25.0–27.0), whereas for women with normal or low-grade cytology, but HPV16 and/or HPV18 positive status, the corresponding estimate was 25.0% (95% CI, 23.3–26.8). Other high-risk HPV genotypes and HPV negative status were associated with lower risks (5-year cumulative incidence 15.5% (95% CI, 14.5–16.6) and 8.2% (95% CI, 7.1–9.5), respectively). Detection of CIN3+ was substantial (cumulative incidence 5.7% (95% CI, 5.4–6.0) and 12.7% (95% CI, 12.2–13.1) after 1 and 5 years, respectively), and overall, cumulative incidence of treatment was 15.2% (95% CI, 14.7–15.7) after 5 years, following similar patterns as observed for CIN2+.

**Conclusions:**

A differentiation of follow-up guidelines by index cytology and HPV16/18 status for women diagnosed with CIN1, might be warranted.

## Introduction

Cervical carcinogenesis is considered a pathological continuum initiated by persistent human papillomavirus (HPV) infection, followed by the development of cervical intraepithelial neoplasia (CIN) grade 1, 2 and 3 and finally invasive cervical cancer [1]. The persistence of carcinogenic HPV genotypes predicts the risk of cervical cancer development, where the highest risk is associated with HPV16 infections [2,3].

There are different recommendations on control intervals and how to manage CIN1 lesions in national screening programs in high-income countries [4–7]. CIN1 reflects an ongoing HPV-infection [8] and is often managed conservatively by active surveillance by repeated cytology and HPV-testing, due to the high regression rate and the low progression rate to more severe premalignant lesions or cancer [9–11]. Excision of CIN1 lesions is, however, considered acceptable in some countries, but increases the risk of preterm birth [12–14]. Considering the low risk of progression of CIN1, excision may to some extent be over-treatment. Furthermore, frequent controls represent a burden on the women and the health care system.

Most previous studies on outcomes in women with CIN1 include only a couple of hundred participants, and very few studies have observation time beyond five years [11]. The evidence-base for guidelines on management of CIN1 in a population setting is therefore uncertain.

In this nationwide, population-based cohort study, we estimated the risk of progression and treatment in women with histologically confirmed CIN1 lesions in the Norwegian Cervical Cancer Screening Program (CervicalScreen Norway).

## Methods

### Data sources

Data were collected from national registries and linked at an individual level using the unique 11-digit national identity number assigned to all residents in Norway. Authors did not have access to information that could identify individual participants during or after data collection. The Cancer Registry of Norway has registered information on all incident cancer diagnoses in Norway since it was established in 1952 [15]. The nationwide CervicalScreen Norway was established in 1995 and is organized by the Cancer Registry of Norway, sending reminders to women aged 25–69 years to attend cytology-based screening every 3^rd^ year [16]. HPV-testing for 14 high-risk HPV-types in samples with low-grade cytology started in 2005, and test results are recorded in the HPV Registry (2005). Since 2015, there has been a gradual shift towards HPV based screening every 5^th^ year for women aged 25–69 years. CervicalScreen Norway receives mandatory reports on cervical samples from all pathology and microbiology laboratories, registered in the Cytology Registry (1991) and the Histology Registry (2002), and on treatment procedures from all gynecologists, registered in the CIN Registry (1997). The incidence database at the Cancer Registry of Norway collects data on invasive tumors of all types and is considered almost complete [17]. Data at the Cancer Registry of Norway are linked regularly to data from the National Population Registry and the Norwegian Cause of Death Registry [18], adding data on residency, emigration and death.

The guidelines in CervicalScreen Norway for referral to colposcopy and biopsy has changed over time. All women with high-grade cytology (atypical squamous cells, cannot exclude a high-grade lesion (ASC-H), high-grade intraepithelial lesion (HSIL), atypical glandular cells of uncertain significance (AGUS) and adenocarcinoma *in situ* (AIS)) have been referred. Women with repeated low-grade cytology over 6 months (low-grade intraepithelial lesion (LSIL), atypical squamous cells of uncertain significance (ASC-US)), or repeated HPV-positivity over 6 months with cytology negative for intraepithelial lesion or malignancy (NILM), have been referred [19]. If the biopsy result is negative histology or CIN1, women are usually recommended follow-up surveillance with cytology and HPV-testing within 6–12 months. If progression is confirmed, excision is normally recommended. Upon regression (HPV negative/NILM), women return to regular screening intervals.

### Study population and variables

**Fig 1:**
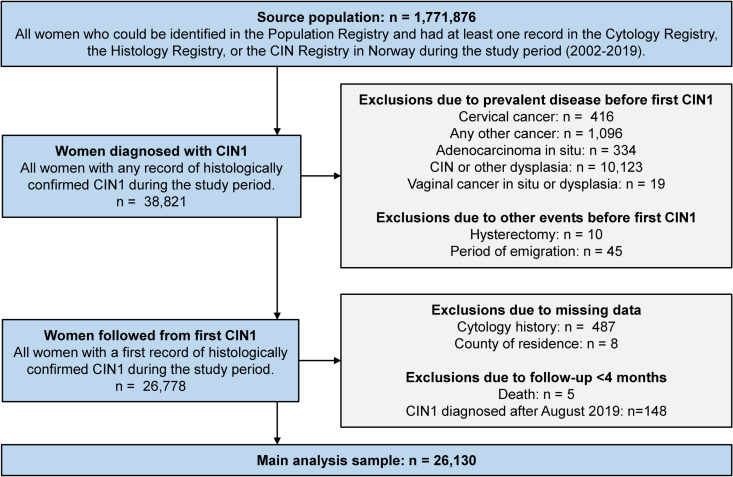
Selection of the study population. CIN – Cervical intraepithelial neoplasia.

The study population was drawn from all women registered in CervicalScreen Norway with at least one cervical screening test from January 1^st^, 2002 until December 31^st^, 2019 (Fig 1), when data from the Histology Registry were available. Among these, 38,821 women were registered with CIN1 in the Histology Registry. In total, 12,691 women with a record of CIN1 (32.7%) were excluded, mainly due to prevalent unspecified CIN or other genital dysplasia before their first CIN1 lesion. Only 495 women with incident CIN1 (1.3%) were excluded due to missing data on cytology history and county of residence, and 153 were excluded due to follow-up time shorter than four months. Thus, the main analyses were based on 26,130 women with histologically confirmed, incident CIN1. A national school-based HPV vaccination program for girls aged 12 years was implemented in 2009, and the first vaccinated birth cohort entered the cervical screening in 2022. Hence, the vast majority of women in this study were unvaccinated.

The primary outcome was histologically verified CIN2, CIN3, AIS, or invasive cervical carcinoma (hereafter referred to as ‘CIN2+’), diagnosed or occurring at least 4 months after the first CIN1 diagnosis. The 4 months’ cutoff was set to account for the registration practice in the Cancer Registry in Norway, where all reports within 4 months are considered as part of the same event. A report of CIN1 which is followed by a report of CIN2+ within 4 months, will therefore be considered as CIN2+. CIN2+ was chosen as the primary outcome, because CIN2 is the threshold for treatment in most cases. However, CIN3 represents more unambiguously premalignant lesions and hence, we included CIN3, AIS, or invasive cervical carcinoma (hereafter referred to as ‘CIN3+’) as a secondary outcome. Another secondary outcome was treatment, defined as either excision of a part of the cervix, with or without detection of CIN2+ on subsequent histological examination, or ablative treatment, with no subsequent histology.

Age at diagnosis was categorized as <30, 30–49 and ≥50 years. Women’s cytology history was obtained from the Cytology Registry, reported according to the 2001 Bethesda System [20]. Inadequate tests were excluded from further analyses. We categorized cytology results as ‘normal or low-grade’ (NILM, NILM with persistent high-risk HPV (hrHPV) infection, LSIL and ASC-US) *versus* ‘high-grade’ (ASC-H, HSIL, AGUS and AIS), allowing up to 4 months delay in reporting cytology results after CIN1 diagnosis. We restricted cytology results before CIN1 diagnosis (index cytology) to tests performed less than 3.5 years before diagnosis of CIN1. If more than one cytology result was available, the most abnormal result was used. We used HPV test results at or before the diagnosis of CIN1, including information on reported genotypes and analysis platform for each analysis, to further categorize samples without high-grade cytology as ‘HPV-’ (no hrHPV detected), ‘HPV16/18+’ (HPV16 and/or HPV18 positive) or ‘HPV other+’ (hrHPV positive, but HPV16 and HPV18 negative). The latter two categories were restricted to results from HPV platforms capable of separating HPV16 and HPV18 from other high-risk HPV genotypes, as outlined in S1 Table.

### Statistical analyses

Women were followed from 4 months after their first CIN1 diagnosis until the date of CIN2+, cancers other than cervical cancer, death from other causes, emigration, or end of follow-up (December 31^st^, 2019). The 4 months’ delay was introduced to avoid immortal follow-up time since registration practice made a diagnosis of CIN2+ within 4 months impossible. For women in the screening age range, more than 3.5 years without samples was considered as program drop-out, and for these women follow-up ended at 3.5 years after the last cytology or histology registered (S1 Fig).

We estimated the cumulative incidence of progression or treatment using a non-parametric competing risk approach with time-dependent weights, where death from causes other than cervical cancer (n = 337), cancers other than cervical cancer (n = 525) and drop-outs (n = 5353) were treated as separate competing events [21]. Cumulative incidence curves were estimated across the full duration of follow-up, overall and according to cytology and HPV status, with tabulation of estimates at 3 and 5 years. To account for the impact of age at diagnosis and county of residence, we used Cox proportional hazards regression to estimate hazard ratios (HRs) of progression or treatment, with censoring at diagnosis of cancers other than cervical cancer, death from causes other than cervical cancer, emigration (n = 239), drop-out or end of follow-up, and adjustment for age (if applicable) and county of residence. Because treatment generally does not eliminate the risk of progression, treatment was not considered a competing risk or reason for censoring in the analyses. The exception was hysterectomy, which was a censoring event when not accompanied by a CIN2+ diagnosis (n = 65). The proportional hazards assumption was checked by visual inspection of log-minus-log plots, and no clear violations were found. Precision was estimated as 95% confidence intervals (CIs).

To investigate the influence of screening program compliance, we repeated analyses of progression to CIN2+ with censoring at 1 year for women with no cytology control registered, in addition to the previously described censoring at 3.5 years. We also repeated analyses of progression to cervical cancer without taking program drop-out into account (i.e., follow-up to cervical cancer, other cancer, death from causes other than cervical cancer, emigration, or end of follow-up). The rationale for this was that drop-out could increase the risk of cancer, which would be captured in the incidence database regardless of program compliance.

### Ethics approval and data availability

The study was approved by the Regional Committee for Medical and Health Research Ethics, South-East Region, Norway (**2017/1910**). Data were accessed for research purpose 01/09/2020. The need for consent was waived by the ethics committee. The study was performed in accordance with the Declaration of Helsinki.

Data were used under license for the current study and are not publicly available in accordance with Norwegian data protection and health research laws. Data may be requested from the data custodian (the Norwegian Institute of Public Health) following approval by the Regional Committee for Medical and Health Research Ethics, as well as the data custodian.

## Results

### Descriptive statistics

The median age at CIN1 diagnosis was 33 years (interquartile range [IQR] 27–45) and median follow-up was 3.6 years (IQR 1.8–6.2). For women diagnosed with CIN2+ during follow-up, median time to detected progression was 1.3 years (IQR 0.8–2.4). Characteristics of the included women are described in Table 1. Overall, 32.6% had a high-grade cytology preceding histological CIN1, with proportions increasing slightly with age. When CIN1 was preceded by normal or low-grade cytology, HPV was present in 75.1%, whereas 12.4% were negative and 12.5% were not examined for HPV. Prevalence of HPV16/18 decreased with increasing age, whereas other high-risk genotypes increased concordantly.

**Table 1:** Cohort characteristics by cytology in 26,130 women diagnosed with index CIN1 in CervicalScreen Norway, 2002–2019.

Index cytology ^1^	Age at diagnosis of CIN1						Total	
	<30 years		30-49 years		≥50 years			
Number of participants (% in columns)	9,997	38.3	11,742	44.9	4,391	16.8	26,130	100
**High grade cytology, n (% in rows)**	**3,092**	**30.9**	**3,846**	**32.8**	**1,568**	**35.7**	**8,506**	**32.6**
ASC-H	1,782	17.8	2,032	17.3	913	20.8	4,727	18.1
HSIL	1,159	11.6	1,394	11.9	403	9.2	2,956	11.3
AGUS	140	1.4	389	3.3	242	5.5	771	3.0
AIS	11	0.1	31	0.3	10	0.2	52	0.2
**Normal or low-grade cytology, n (% in rows)**	**6,905**	**69.1**	**7,896**	**67.2**	**2,823**	**64.3**	**17,624**	**67.4**
Normal	685	6.9	1,292	11.0	1,097	25.0	3,074	11.8
LSIL	2,008	20.1	2,116	18.0	445	10.1	4,569	17.5
ASC-US	4,212	42.1	4,488	38.2	1,281	29.2	9,981	38.2
HPV status and genotype								
HPV −	660	9.6	985	12.5	543	19.2	2,188	12.4
HPV+	5,457	79.0	6,202	78.5	1,567	55.5	13,226	75.1
*HPV16/18+* ^2^	*1,924*	*35.3*	*1,735*	*28.0*	*348*	*22.2*	*4,007*	*30.3*
*Other HR+* ^3^	*2,624*	*48.1*	*3,605*	*58.1*	*1,040*	*66.4*	*7,269*	*55.0*
*No genotype testing*	*909*	*16.6*	*862*	*13.9*	*179*	*10.4*	*1,950*	*14.7*
Unknown HPV status	788	11.4	709	9.0	713	25.3	2.204	12.5

^1^Cytology registered within the previous 3.5 years before CIN1 diagnosis. If more than one cytology is registered, it refers to the most abnormal cytology within these 3.5 years.

^2^HPV 16 and/or HPV 18 positive.

^3^High-risk HPV positive, but HPV 16 and HPV 18 negative.

Abbreviations: AGUS – atypical glandular cells of uncertain significance, AIS – adenocarcinoma *in situ*, ASC-H – atypical squamous cells, cannot exclude a high-grade lesion, ASC-US – atypical squamous cells of uncertain significance, CIN – Cervical intraepithelial neoplasia, CIN1 – CIN grade 1, CIN2+ – CIN grade 2 or worse, CIN3+ – CIN grade 3 or worse, HPV – human papilloma virus, HR – high risk, HSIL – high-grade intraepithelial lesion, LSIL – low-grade intraepithelial lesion.

### Progression of histologically confirmed CIN1

**Fig 2:**
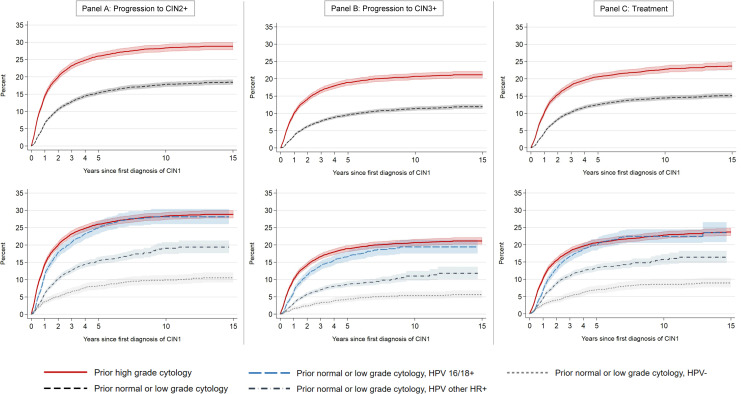
Cumulative incidence of progression to CIN2+ (Panel A, left column), progression to CIN3+ (Panel B, mid column), and treatment (Panel C, right column) following an index CIN1 diagnosis in CervicalScreen Norway 2002-2019, according to index cytology (top figure in each panel) and combinations of index cytology and HPV genotype status (bottom figure in each panel) preceding the index CIN1 diagnosis. Abbreviations: CIN – cervical intraepithelial neoplasia, HPV – human papilloma virus. CIN2+ includes CIN2, CIN3, adenocarcinoma *in situ* (AIS) and cervical cancer. CIN3+ includes CIN3, AIS and cervical cancer. Treatment includes excision and ablation. HPV 16/18+ - HPV test positive for genotypes 16 and/or 18. HPV other HR+ - HPV test positive for HPV high risk genotypes other than 16 and 18 (i.e., HPV 31, 33, 35, 39, 45, 51, 52, 56, 58, 59, 66, and 68). HPV- - HPV test negative. Shaded areas indicate 95% confidence intervals.

Among 26,130 included women with CIN1, 4,491 were diagnosed with CIN2+ during follow-up. Overall, the cumulative incidence of CIN2+ increased mainly during the first years, reaching 9.1% within 1 year and 19.0% within 5 years and thereafter levelling off (Table 2 and S2 Fig). CIN2+ cumulative incidence curves according to cytology history showed similar patterns, with the highest increase in risk the first years after diagnosis, before levelling off after around 5 years (Fig 2, Panel A). Women with prior high-grade cytology had substantially higher incidence of CIN2+ than women with normal or low-grade cytology (26.0% vs 15.4% within 5 years) (Table 3). Further stratification of the latter group by HPV genotype indicated that in HPV16/18+ women, risk approached that seen for high-grade cytology (25.0%), whereas HPV other+ and HPV- status was associated with lower risks (15.5% and 8.2%, respectively). Progression was detected twice as often among younger women (21.3% vs 10.9% within 5 years for women <30 and ≥50 years, respectively). Results from Cox regression across the entire follow-up period were consistent with the cumulative incidence patterns, with little influence of adjustment for age and county (Table 3). Consistent with patterns of cumulative incidence, hazard rates of CIN2+ were high during the first year of follow-up, then declined before levelling off around 5 years after diagnosis of CIN1 (S3 Fig). Sensitivity analyses with censoring of women who missed the initial cytology control at 1 year, showed slightly lower cumulative incidence estimates, but associations with prognostic factors for progression (age, cytology history and HPV status) were similar to the main analyses (S2 Table and S4 Fig).

**Table 2:** Cumulative incidence of progression and treatment following an index CIN1 diagnosis in CervicalScreen Norway 2002-2019, by time since diagnosis.

Outcome	Cumulative incidence (95% CI)				
	1 year	3 years	5 years	10 years	15 years
Progression to CIN2+	9.1 (8.8-9.5)	16.3 (15.8-16.8)	19.0 (18.4-19.5)	21.4 (20.8-22.0)	21.9 (21.3-22.6)
Progression to CIN3+	5.7 (5.4-6.0)	10.7 (10.3-11.1)	12.7 (12.2-13.1)	14.5 (14.0-15.1)	15.1 (14.6-15.7)
Treatment	6.2 (5.9-6.6)	12.9 (12.4-13.3)	15.2 (14.7-15.7)	17.2 (16.7-17.8)	18.1 (17.4-18.7)

Abbreviations: CI - confidence interval, CIN – Cervical intraepithelial neoplasia, CIN1 – CIN grade 1, CIN2+ – CIN grade 2 or worse, CIN3+ – CIN grade 3 or worse

**Table 3:** Cumulative incidence and hazard ratios of progression and treatment following an index CIN1 diagnosis in CervicalScreen Norway 2002-2019, by patient and lesion characteristics.

Outcome	Patient and lesion characteristics		Events	Patients	Cumulative incidence (95% CI)		Hazard ratio ^1^ (95% CI)	
					3 years	5 years	Unadjusted	Adjusted ^2^
**Progression to CIN2+**	Age	<30 years	1,916	9,997	18.3 (17.5-19.1)	21.3 (20.4-22.2)	1	1
		30-39 years	1,317	6,808	18.3 (17.3-19.3)	21.6 (20.5-22.7)	1.02 (0.95-1.10)	1.02 (0.95-1.09)
		40-49 years	814	4,934	15.8 (14.7-16.9)	18.1 (16.9-19.3)	0.84 (0.77-0.91)	0.84 (0.77-0.91)
		50-59 years	296	2,735	10.4 (9.3-11.7)	11.8 (10.5-13.2)	0.53 (0.47-0.60)	0.54 (0.48-0.61)
		≥60 years	148	1,656	8.0 (6.8-9.5)	9.5 (8.1-11.2)	0.43 (0.37-0.51)	0.44 (0.37-0.52)
	Index cytology	Normal or low-grade	2,395	17,624	12.9 (12.3-13.4)	15.4 (14.8-16.0)	1	1
		High-grade	2,096	8,506	23.2 (22.3-24.2)	26.0 (25.0-27.0)	1.85 (1.74-1.96)	1.94 (1.83-2.06)
	Index cytology & HPV ^3^	Normal or low-grade, HPV -	200	2,263	6.6 (5.7-7.8)	8.2 (7.1-9.5)	0.56 (0.48-0.66)	0.60 (0.51-0.70)
		Normal or low-grade, HPV other HR+	884	7,269	12.7 (11.9-13.6)	15.5 (14.5-16.6)	1	1
		Normal or low-grade, HPV 16/18+	739	4,007	20.8 (19.4-22.3)	25.0 (23.3-26.8)	1.71 (1.55-1.89)	1.63 (1.48-1.80)
		High-grade	2,096	8,506	23.2 (22.3-24.2)	26.0 (25.0-27.0)	1.91 (1.77-2.07)	2.02 (1.87-2.19)
**Progression to CIN3+**	Age	<30 years	1,313	9,997	12.2 (11.5-12.9)	14.7 (13.9-15.5)	1	1
		30-39 years	929	6,808	12.6 (11.8-13.5)	15.1 (14.2-16.1)	1.05 (0.96-1.14)	1.05 (0.96-1.14)
		40-49 years	515	4,934	10.0 (9.1-10.9)	11.4 (10.5-12.4)	0.77 (0.70-0.86)	0.79 (0.71-0.87)
		50-59 years	165	2,735	5.9 (5.0-6.9)	6.7 (5.7-7.8)	0.44 (0.37-0.52)	0.46 (0.39-0.54)
		≥60 years	85	1,656	4.6 (3.6-5.7)	5.3 (4.3-6.6)	0,37 (0.30-0.46)	0.39 (0.32-0.49)
	Index cytology	Normal or low-grade	1,488	17,624	7.8 (7.4-8.2)	9.5 (9.1-10.1)	1	1
		High-grade	1,519	8,506	16.6 (15.8-19.8)	18.9 (18.1-19.8)	2.11 (1.96-2.26)	2.26 (2.11-2.43)
	Index cytology & HPV ^3^	Normal or low-grade, HPV -	106	2,263	3.4 (2.7-4.2)	4.3 (3.5-5.3)	0.53 (0.43-0.66)	0.59 (0.48-0.73)
		Normal or low-grade, HPV other HR+	496	7,269	7.1 (6.4-7.7)	8.7 (7.9-9.5)	1	1
		Normal or low-grade, HPV 16/18+	487	4,007	13.9 (12.7-15.2)	16.6 (15.2-18.2)	1.98 (1.75-2.25)	1.87 (1.65-2.12)
		High-grade	1,519	8,506	16.6 (15.8-19.8)	18.9 (18.1-19.8)	2.41 (2.18-2.68)	2.59 (2.34-2.87)
**Treatment**	Age	<30 years	1,303	9,923	12.2 (11.5-12.9)	14.8 (14.0-15.6)	1	1
		30-39 years	1,021	6,687	14.2 (13.3-15.1)	17.0 (16.0-18.1)	1.19 (1.09-1.29)	1.19 (1.10-1.30)
		40-49 years	733	4,799	14.5 (13.5-15.6)	16.8 (15.7-18.0)	1.17 (1.07-1.28)	1.19 (1.09-1.30)
		50-59 years	289	2,614	10.9 (9.7-12.2)	11.8 (10.5-13.2)	0.83 (0.73-0.94)	0.87 (0.77-0.99)
		≥60 years	163	1,585	10.0 (8.5-11.7)	11.2 (9.6-13.0)	0.77 (0.65-0.90)	0.81 (0.69-0.96)
	Index cytology	Normal or low-grade	1,895	17,359	10.3 (9.8-10.8)	12.5 (12.0-13.1)	1	1
		High-grade	1,614	8,256	18.1 (17.2-19.0)	20.6 (19.7-21.5)	1.77 (1.66-1.90)	1.84 (1.72-1.97)
	Index cytology & HPV ^3^	Normal or low-grade, HPV -	166	2,226	5.3 (4.4-6.3)	6.9 (5.9-8.2)	0.55 (0.46-0.65)	0.56 (0.47-0.66)
		Normal or low-grade, HPV other HR+	742	7,155	11.0 (10.2-11.8)	13.3 (12.4-14.3)	1	1
		Normal or low-grade, HPV16/18+	561	3,947	16.7 (15.3-18.1)	20.0 (18.4-21.7)	1.52 (1.36-1.69)	1.50 (1.35-1.68)
		High-grade	1,614	8,256	18.1 (17.2-19.0)	20.6 (19.7-21.5)	1.70 (1.56-1.86)	1.75 (1.61-1.91)

^1^Estimated over the full follow-up period.

^2^Adjusted for age (if applicable) and county

^3^HPV positive cases with no genotyping are excluded, other+ refers to HPV 31, 33, 35, 39, 45, 51, 52, 56, 58, 59, 66, and 68.

Abbreviations: CI - confidence interval, CIN – Cervical intraepithelial neoplasia, CIN1 – CIN grade 1, CIN2+ – CIN grade 2 or worse, CIN3+ – CIN grade 3 or worse, HPV – human papilloma virus, HR – high risk.

There were 3,007 cases of progression to CIN3+, with an overall cumulative incidence of 12.7% within 5 years (Table 2). Patterns according to age, cytology history and HPV status were similar to those seen for CIN2+ (Table 3, Fig 2, Panel B and S3 Fig). Among women with CIN1 who attended CervicalScreen Norway with ≤3.5 years between samples, 54 cervical cancers were detected across the full study period, corresponding to a cumulative incidence of 0.30% (95% CI 0.22–0.40)(S2 Fig). These were diagnosed after a median follow-up of 1.7 years (IQR 1.0–4.2), the majority after high-grade index cytology (37 women, 68.5%). Altogether, 27 cases were diagnosed with stage I disease (50%), 7 cases with stage II or III (13%), and 20 women with unknown stage (37%).

### Treatment

Altogether, 4,031 women were treated, where 522 women had their first ever CIN1 diagnosis in cone specimen and were excluded from treatment analyses, leaving 3,509 women treated during follow-up. The overall cumulative incidence of treatment was 15.2% after 5 years (Table 2 and S2 Fig), i.e., lower than the incidence of CIN2+, but higher than CIN3+. Cumulative incidence curves for treatment showed similar patterns as the progression curves, with the highest increase seen during the first 3–5 years. A substantially higher proportion of women with high-grade cytology or HPV16/18+ with normal or low-grade cytology was treated, compared to women with normal or low-grade cytology HPV- or HPV other+ (Table 3 and Fig 2, Panel C). Women aged 30–49 years at CIN1 diagnosis had a higher occurrence of treatment than younger and older women (Table 3). For women <30 years at detection of CIN1, treatment cumulative incidence (12.2 and 14.8% at 3 and 5 years, respectively) was somewhat lower than the incidence of CIN2+, but comparable to the incidence of CIN3+. In contrast, among the oldest women, cumulative incidence of treatment (10.5%) was twice that of CIN3+ (5.4%). Adjustment for age and county did not change the associations between cytology and HPV status and occurrence of treatment (Table 3), and hazard rates followed similar patterns as for progression (S3 Fig).

## Discussion

To our knowledge, our study is the largest nationwide population-based, long-term cohort study, providing estimates of risk of progression (CIN2+) and treatment following histologically confirmed CIN1 with a follow-up time up to 18 years. In the context of CervicalScreenNorway, the overall risk of detecting CIN2+ among women with CIN1 was close to 20% within 5 years. Younger age, high-grade index cytology and HPV16/HPV18 positivity were associated with substantially higher risk of progression to CIN2+ and CIN3+ compared to NILM and low-grade cytology positive hrHPV types other than HPV16/18.

In our study, one third of index cytology samples were high-grade, in contrast to most studies on CIN1 where almost all index cytology samples were low-grade [7,11], with a reported lower risk of progression. A comparably high progression risk of 24% has been demonstrated in previous smaller studies, with a high proportion of high-grade index cytology [22,23]. A recent systematic review of natural history of CIN, including 63 studies with a total of 8,767 participants, reported considerable heterogeneity between studies according to sample size, follow-up, index cytology and histologically confirmed CIN1 diagnosis [11]. Most studies had from 6 to 54 months follow-up. In total, 11% of CIN1 progressed to CIN2+, 2% to CIN3+ and 0.03% to cervical cancer [11]. The differences in risk of progression between the studies may be due to differences in follow-up time and considerably different proportion in high-grade index cytology. Delayed or missed detection of prevalent CIN2+ might result from sampling error and interobserver variation in colposcopy [24] and pathology examination [25]. According to national Norwegian guidelines, biopsies should be obtained from any lesion detected at colposcopy, and from all quadrants if the colposcopy is normal according to national guidelines [5].

Consensus guidelines from American Society for Colposcopy and Cervical pathology in 2019 [6] are based on risk-estimates from 48,004 women with CIN1 registered at Kaiser Permanente Northern California (KPNC). The 5-year risk of CIN3+ in women with HPV positive NILM or low-grade cytology *versus* high-grade cytology was 2.3–2.8% and 3.8–6.5%, respectively, much lower than our estimates and may reflect higher sensitivity for diagnosing abnormalities, for referral to colposcopy and treatment in the US, but also a study population of relatively healthy women at low risk [7]. The authors commented that risk-estimates after CIN1 with high-grade index cytology were less reliable, as they were rare, with an inherently high risk of occult disease that should be managed carefully.

An Italian study including 434 women with CIN1 with adequate colposcopy and mainly low-grade index cytology, detected CIN2–3 in 7.4%, CIN3 in 0.9% and no cancers during 5 years of follow-up. The authors concluded that HPV status and index cytology should be included in the assessment together with colposcopic impression in women with CIN1 [26].

Although HPV16 confers the highest risk of developing CIN and cervical cancer, HPV types such as HPV18 and HPV33 also increases risk of progression compared to other hrHPV types [3]. A recent study from KPNC demonstrated that HPV16 and 18 positivity at screening increased the immediate and 5-years risk of CIN3+ substantially, leading to recommendations of referral for colposcopy even in women with NILM [27]. This is comparable to our results as HPV16/18 positivity conferred a doubled risk of progression to CIN2+ compared to HPV other+. Based on our results, yearly observation of CIN1 lesions in women with low-grade cytology negative for HPV16 and/or HPV18 seems acceptable, and justifiable even for women with high-grade index cytology or HPV16 and/or HPV18 positivity, although a shorter interval might be appropriate for the latter group.

Older women in our study had a lower risk of progression, despite similar distributions of index cytology results in younger and elderly women. Contrary to our findings, progression to CIN2+ and CIN3+ in HPV-positive women is reported to increase with age [28,29]. For newly detected infections, progression was not higher in older compared to younger women, but persistent infections were more frequent among the oldest [28]. Persistent HPV infections, particularly HPV16, are associated with progression [3]. In our cohort, a substantially lower proportion of older women were HPV16/18 positive, which might explain their lower progression rate compared to younger women. Also, older women would have had more screening rounds before the start of the study period, and hence, a first diagnosis of CIN1 might more often represent a recently developed lesion.

The occurrence of treatment corresponded well with estimates of progression, and hence provided no support for large extents of non-indicated treatment. To validate treatment registration in the CIN Registry, we obtained aggregated data on ablations and surgical treatment during 2008–2021 from the Norwegian Patient Registry, where diagnoses and procedures from all activity in specialist health care nationally are recorded since 2008 [30]. These data indicated under-reporting of ablation in the CIN Registry, where only 24 of 3,509 registered treatments between 2002 and 2019 were ablations, as opposed to 153 (5.9%) of 2,610 treatments registered in the Norwegian Patient Registry (S3 Table).

Treatment of CIN2 will influence the risk of developing CIN3+. Because treatment threshold is at CIN2+, our analyses of risk of progression to CIN3+ must be interpreted considering clinical practice where CIN2 lesions are removed. We believe our analyses of CIN3+ should be seen as an indication of how many of the progressing CIN1 lesions that have reached CIN3+ before being diagnosed, rather than the risk of progression to CIN3+ in a hypothetical scenario where treatment indication is CIN3+.

Finally, we expect that HPV vaccination has not substantially influenced progression and treatment rates of CIN1 in this study. Nationwide school-based HPV vaccination was implemented for 12-year-old girls in Norway in 2009. The oldest vaccinated birth cohort was therefore just 22 years at the end of the study period. Only very few women in earlier birth cohorts would have received the vaccine at their own initiative. Moreover, herd immunity in the vaccinated birth cohorts seems to provide limited protection in older birth cohorts [31]. With HPV vaccinated birth cohorts entering the screening program, the risk of progression from CIN1 might change. Although a wide range of HPV types can lead to CIN1, the HPV types covered by the vaccines show the highest rates of progression to higher-grade lesions such as CIN2, CIN3 and, ultimately, to cervical cancer [32]. HPV vaccination prevents infection with these HPV types [33–36]. As a result, CIN1 lesions detected in a vaccinated population may be expected to more often be caused by less oncogenic HPV types. Further research is needed to determine the optimal management of CIN1 in HPV-vaccinated populations and ensure that clinical recommendations and public health guidelines remain evidence-based.

### Strengths and limitations

The strengths of our study include the population-based design and the large number of women in a wide age range, in the context of a national screening program. The long-follow-up (up to 18 years) allowed estimation of both short and long-term risk of progression. Mandatory reporting ensured nearly complete data on cytology and histology results. With linkage of individual level data, loss-to follow-up was limited to screening drop-out and emigration. Moreover, all baseline and follow-up diagnoses were confirmed through histopathology, which increases validity.

Importantly, our study does not describe the natural history of CIN1, nor the risk associated with persistent HPV-infection or persistent CIN1. Women with CIN1 not attending the screening program at least once every 3.5 years after CIN1 diagnosis, were censored, and our results apply to women reasonably compliant with screening guidelines. Screening-attendance in Norway during the study period was suboptimal at 69–76% [18], and under-screened women have an increased risk of CIN2+ and cervical cancer [37,38]. Hence, the steep increase in CIN2+ during the first two years might partly result from long-standing CIN1 with shorter time from detection to progression, or from prevalent high-grade disease missed in the first examination. Despite exclusion of all registered prevalent CIN cases, registration was likely incomplete before the Histology Registry started in 2002, resulting in some prevalent cases appearing as incident cases. Finally, we had no information on factors such as socioeconomic status, lifestyle, and immunosuppression [39–41], which might have been used for additional risk stratification.

## Conclusions

This large, population-based cohort study contributes evidence on the risk of progression of CIN1 in the context of a national screening program. The overall risk of progression reached 20% after 5 years, but differed considerably according to age, HPV type and index cytology. Our results should be used to inform women with CIN1 about risk of progression, and tailored follow-up for women with CIN1 based on HPV genotyping and index cytology results may be warranted.

## Supporting information

S1 Table Overview of the HPV tests applied during the study period 2002–2019. (DOCX)

S2 Table Cumulative incidence and hazard ratios of progression to CIN2+ following an index CIN1 diagnosis in CervicalScreen Norway 2002–2019, according to patient and lesion characteristics. Sensitivity analyses with censoring of women with no cytology control within 1 year.
(DOCX)

S3 Table Treatment modalities performed in Norwegian public hospitals and outpatient clinics in women registered with an index CIN1 in the time-period 2008–2021, according to the Norwegian Patient Registry. (DOCX)

S1 Fig Timeline and analytical decisions during follow-up. (TIFF)

S2 Fig Cumulative incidence of progression and treatment following an index CIN1 diagnosis in CervicalScreen Norway 2002–2019. (TIFF)

S3 Fig Hazard rates of progression and treatment following an index CIN1 diagnosis in CervicalScreen Norway 2002–2019, according to index cytology and HPV genotypes. (TIF)

S4 Fig Cumulative incidence and Hazard rates of progression following an index CIN1 diagnosis in CervicalScreen Norway 2002–2019, according to index cytology and HPV genotypes. Sensitivity analyses with censoring of women with no cytology control within 1 year.
(TIF)
