# Supplementary material for: Cervical intraepithelial neoplasia grade 1 and long-term risk of progression and treatment
Source: PLoS One. 2025 Apr 23;20(4):e0320739. doi: 10.1371/journal.pone.0320739 (PMC12017515; doi:10.1371/journal.pone.0320739)
Supplement: S1 Table — (DOCX) [file pone.0320739.s001.docx]

| **S1 Table. Overview of the HPV tests applied during the study period 2002-2019 in Norway.** | | |
| --- | --- | --- |
| **HPV tests** | **Genotyping** | **High-risk HPV genotypes** |
| Cobas® 4800 HPV test | Yes | 16,18 individually, 12 pooled (31,33,35,39,45,51,52,56,58,59,66,68) |
| Pre-Tect HPV-Proofer | Yes | 16,18,31,33,45 individually |
| Pap Type 13 HPV-test | Yes | 16,18,31,33,35,39,45,51,52,56,58,59,68 individually |
| PAP 13 | Yes | 16,18,31,33,35,39,45,51,52,56,58,59,68,6,11 individually |
| Abbot RealTi*m*e High Risk HPV test | Yes | 16,18 individually, 12 pooled (31,33,35,39,45,51,52,56,58,59,66,68) |
| BD-Onclarity™ HPV Assay | Yes | 16,18,31,45,51,52 individually, by group (33,58) (35,39,68) (56,59,66) |
| Amplicore¹ | No | (included only when performed together with PreTect HPV-Proofer) |
| Hybrid Capture 2² | No | (included only when performed together with an in-house genotyping test) |
| ¹One laboratorium performed genotyping with Pre-Tect HPV-Proofer after Amplicore-tests, and ²one laboratorium performed genotyping with an in-house genotyping test after positive HybridCapture 2.  Abbreviations: HPV – human papilloma virus | | |
