# Supplementary material for: Cervical intraepithelial neoplasia grade 1 and long-term risk of progression and treatment
Source: PLoS One. 2025 Apr 23;20(4):e0320739. doi: 10.1371/journal.pone.0320739 (PMC12017515; doi:10.1371/journal.pone.0320739)
Supplement: S2 Table — Sensitivity analyses with censoring of women with no cytology control within 1 year. (DOCX) [file pone.0320739.s002.docx]

| **S2 Table. Cumulative incidence and hazard ratios of progression to CIN2+ following CIN1 diagnosis in CervicalScreen Norway 2002-2019, according to patient and lesion characteristics. Sensitivity analyses with censoring of women with no cytology control within 1 year.** | | | | | | | | |
| --- | --- | --- | --- | --- | --- | --- | --- | --- |
|  |  |  |  |  | **Cumulative incidence (95% CI)** | | **Hazard ratio ^1^ (95% CI)** | |
| **Outcome** | **Patient and lesion characteristic** | | **Events** | **Patients** | **3 years** | **5 years** | **Unadjusted** | **Adjusted ^2^** |
| **Progression to CIN2+** | Age | <30 years | 1,916 | 9,997 | 16.9 (16.2-17.7) | 19.5 (18.7-20.4) | 1.04 (0.97-1.10) | 1.03 (0.97-1.10) |
|  |  | 30-49 years | 2,131 | 11,742 | 16.2 (15.6-17.0) | 18.6 (17.9-19.4) | 1 | 1 |
|  |  | ≥50 years | 444 | 4,391 | 9.0 (8.2-10.0) | 10.3 (9.4-11.3) | 0.55 (0.49-0.61) | 0.56 (0.50-0.62) |
|  | Index cytology | Normal or low-grade | 2,395 | 17,624 | 12.0 (11.5-12.6) | 14.2 (13.6-14.8) | 1 | 1 |
|  |  | High-grade | 2,096 | 8,506 | 21.9 (21.0-22.8) | 24.2 (23.3-25.2) | 1.82 (1.71-1.93) | 1.91 (1.80-2.03) |
|  | Index cytology & HPV ^3^ | Normal or low-grade, HPV - | 200 | 2,263 | 6.3 (5.3-7.4) | 7.7 (6.6-8.9) | 0.57 (0.49-0.70) | 0.60 (0.51-0.71) |
|  |  | Normal or low-grade, HPV other HR+ | 884 | 7,269 | 11.9 (11.1-12.7) | 14.4 (13.4-15.4) | 1 | 1 |
|  |  | Normal or low-grade, HPV 16/18+ | 739 | 4,007 | 19.2 (17.8-20.6) | 22.9 (21.2-24.6) | 1.70 (1.54-1.88) | 1.63 (1.48-1.81) |
|  |  | High-grade | 2,096 | 8,506 | 21.9 (21.0-22.8) | 24.2 (23.3-25.2) | 1.91 (1.76-2.07) | 2.01 (1.86-2.19) |
| ^1^ Estimated over the full follow-up period. ^2^ Adjusted for age (if applicable) and county ^3^ HPV positive cases with no genotyping are excluded, other+ refers to HPV 31,33,35,39,45,51,52,56,58,59,66,68  Abbreviations: CI - confidence interval, CIN – Cervical intraepithelial neoplasia, CIN1 – CIN grade 1, CIN2+ – CIN grade 2 or worse, HPV – human papilloma virus, HR – high risk | | | | | | | | |
