# Supplementary material for: Cervical intraepithelial neoplasia grade 1 and long-term risk of progression and treatment
Source: PLoS One. 2025 Apr 23;20(4):e0320739. doi: 10.1371/journal.pone.0320739 (PMC12017515; doi:10.1371/journal.pone.0320739)
Supplement: S3 Table — (DOCX) [file pone.0320739.s003.docx]

| **S3 Table. Treatment modalities performed in Norwegian public hospitals and outpatient clinics in women registered with CIN1¹ in the time period 2008-2021, according to the Norwegian Patient Registry.** | | |
| --- | --- | --- |
| **NOMESKO Classification of Surgical Procedures** | **Treatment modalities** | **Number of events**  **(N = 2,610)** |
|  | Excision, all procedures | 2,457 |
| LDB00 | Extirpation of cervical lesion | 126 |
| LDC00 | Cold-knife conization | 0 |
| LDC03 | Electrosurgical or laser excision | 2,323 |
| LDC10 | Resection of cervix | 8 |
|  | Ablation², all procedures | 153 |
| LDB10 | Cryocautery | 67 |
| LDB20 | Thermal coagulation or laser ablation | 86 |
| ¹ Data were drawn from a source population of women diagnosed with N870 CIN1 according to the International Classification of Diseases, 10th Revision. ² If ablative treatments (LDB10 or LDB20) were registered together with excisional treatment (LDB00, LDC03 or LDC10), the event was registered as excision, not ablation. Treatment procedure “other treatment, not specified” (LDW96) was excluded, n=17.  Abbreviations: CIN1 – Cervical intraepithelial neoplasia grade 1, NOMESCO – Nordic Medico-Statistical Committee | | |
