# Supplementary figures and images for: Cervical intraepithelial neoplasia grade 1 and long-term risk of progression and treatment
Source: PLoS One. 2025 Apr 23;20(4):e0320739. doi: 10.1371/journal.pone.0320739 (PMC12017515; doi:10.1371/journal.pone.0320739)

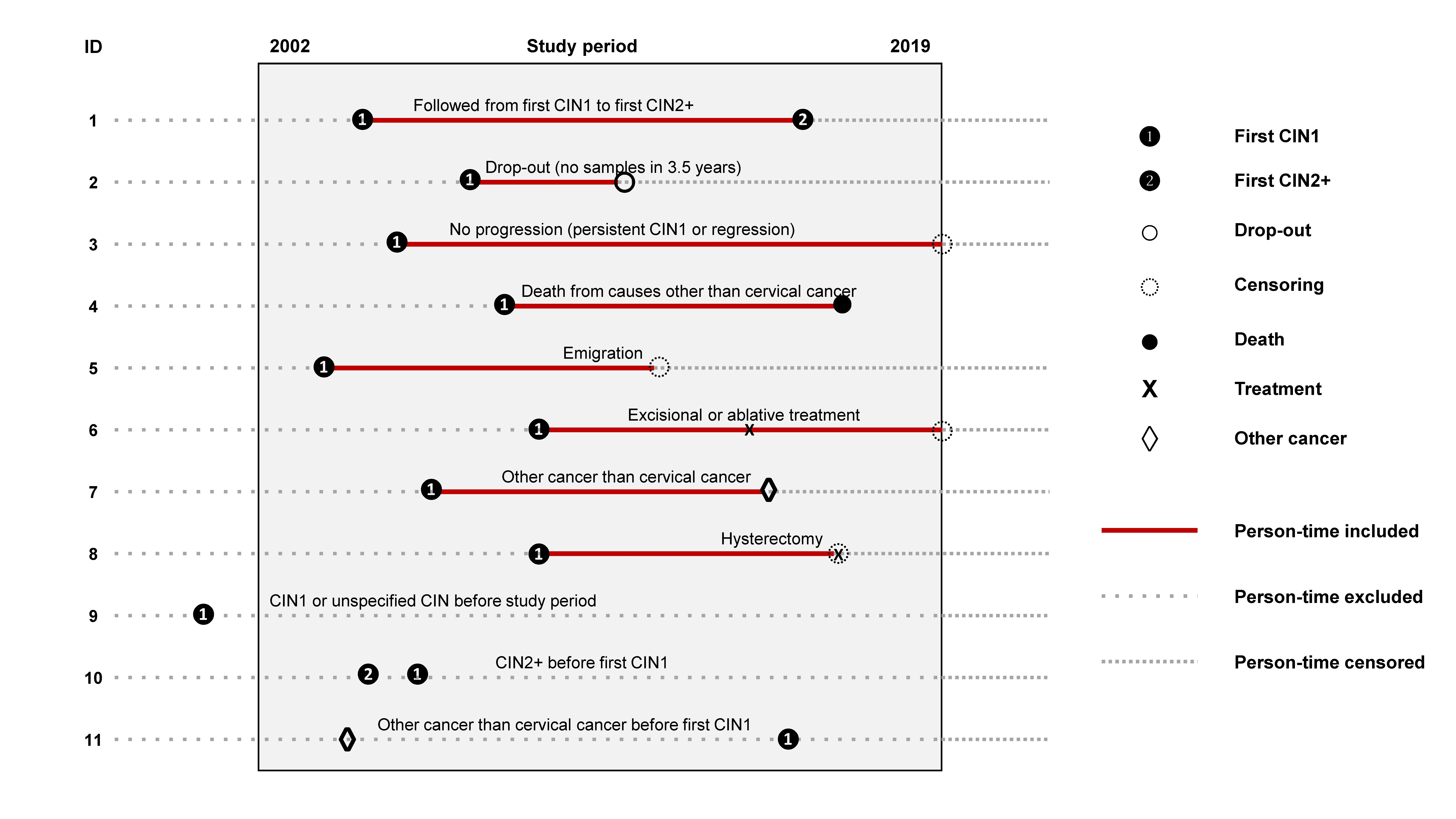

Supplement: S1 Fig — (TIFF) [file pone.0320739.s004.tiff]

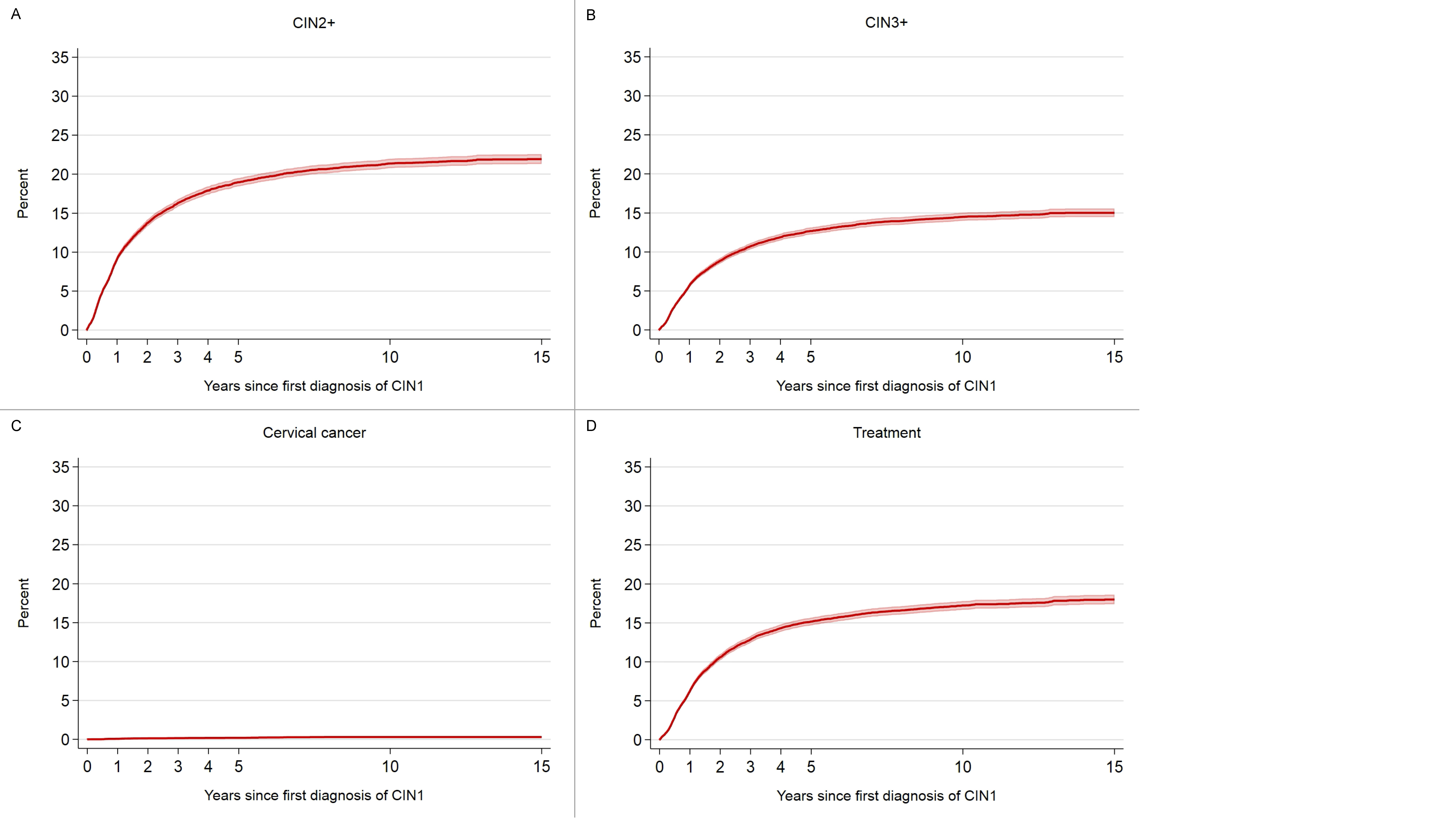

Supplement: S2 Fig — (TIFF) [file pone.0320739.s005.tiff]

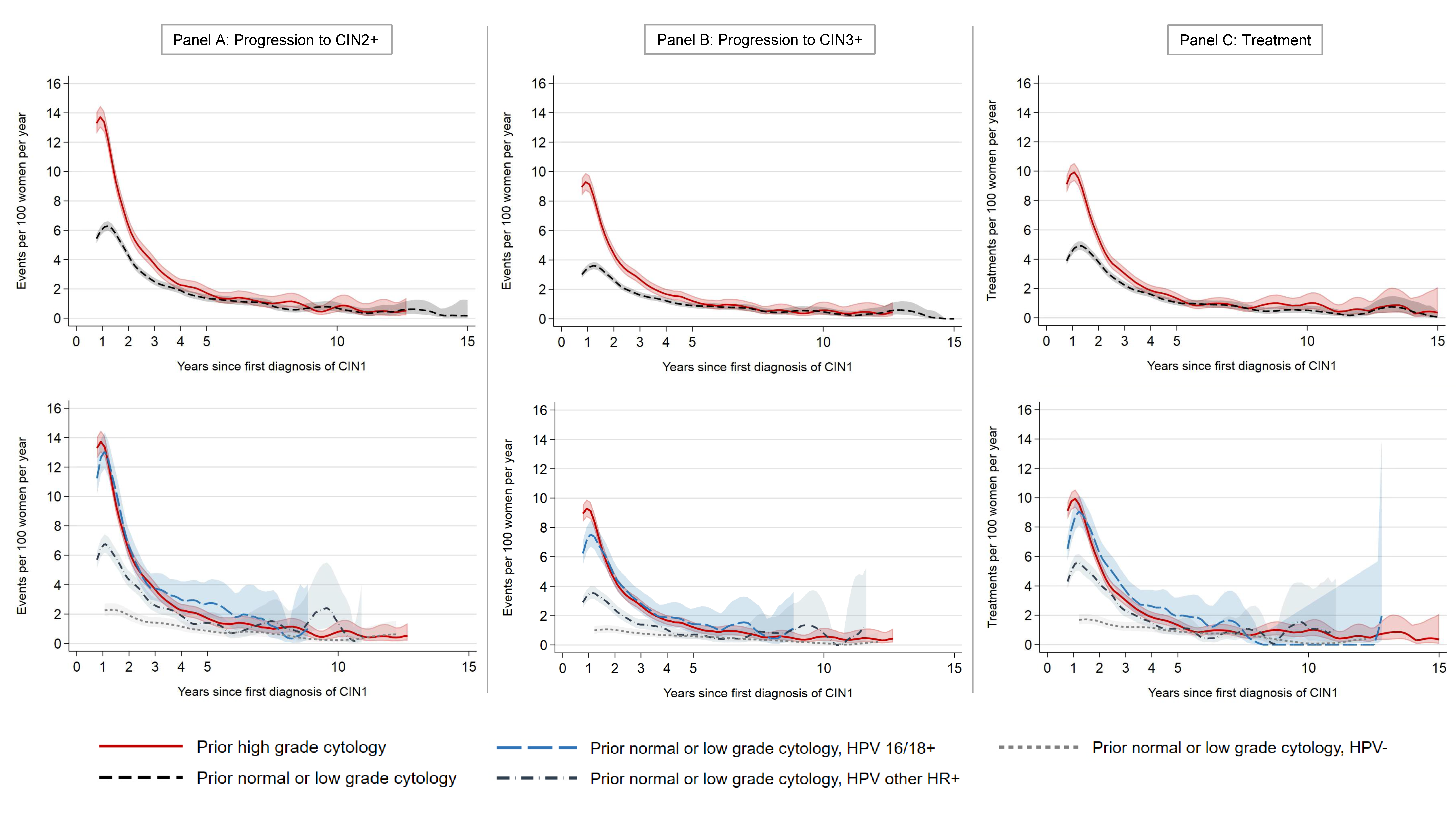

Supplement: S3 Fig — (TIF) [file pone.0320739.s006.tif]

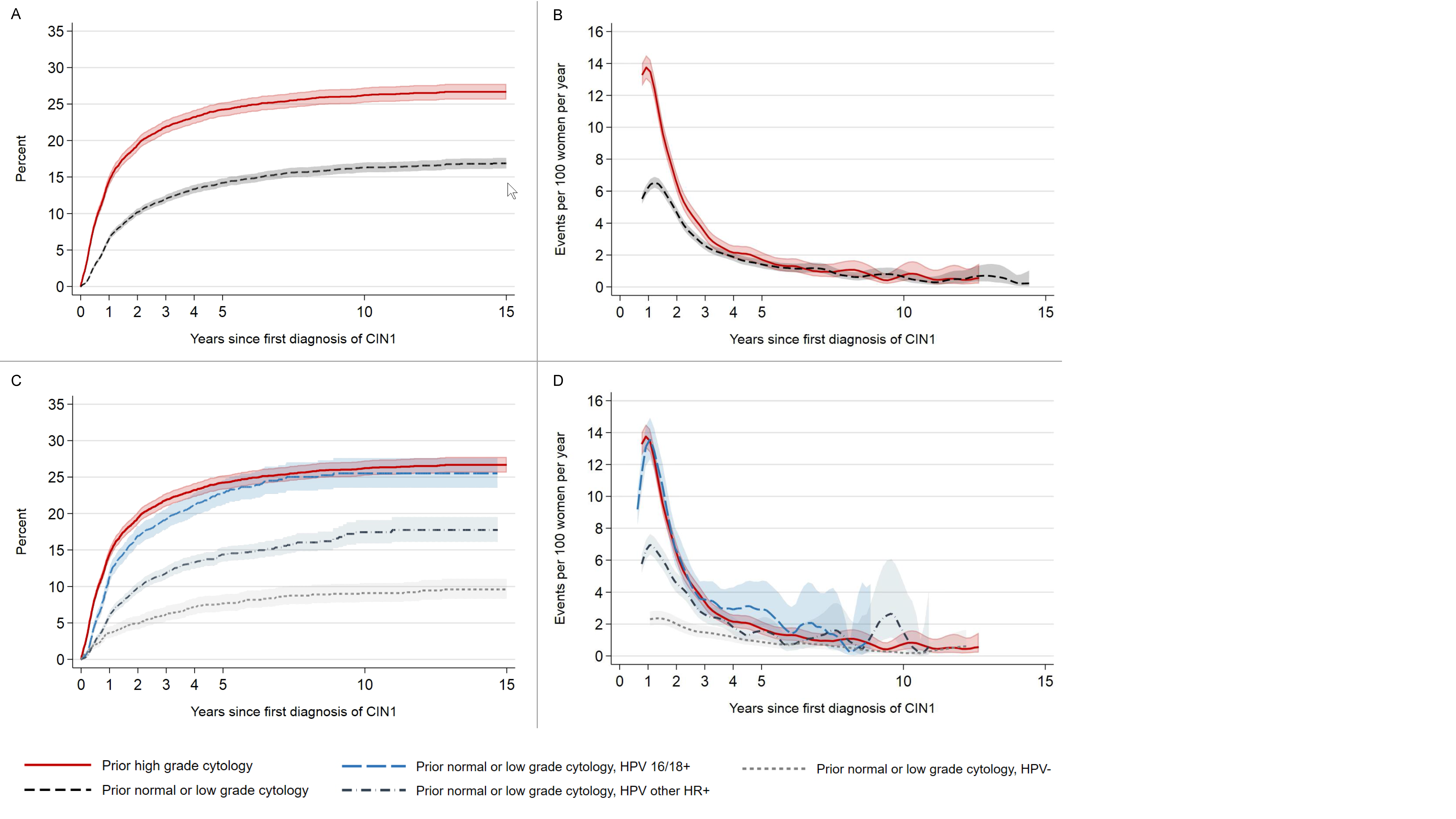

Supplement: S4 Fig — Sensitivity analyses with censoring of women with no cytology control within 1 year. (TIF) [file pone.0320739.s007.tif]
